# Association of socioeconomic status change between infancy and adolescence, and blood pressure, in South African young adults: Birth to Twenty Cohort

**DOI:** 10.1136/bmjopen-2015-008805

**Published:** 2016-03-30

**Authors:** Juliana Kagura, Linda S Adair, Pedro T Pisa, Paula L Griffiths, John M Pettifor, Shane A Norris

**Affiliations:** 1 MRC/Wits Developmental Pathways for Health Research Unit, Department of Paediatrics and Child Health, Faculty of Health Sciences, University of the Witwatersrand, Johannesburg, Gauteng, South Africa; 2 Department of Nutrition, University of North Carolina, Chapel Hill, North Carolina, USA; 3 Centre for Global Health and Human Development, School of Sport, Exercise, and Health Sciences, Loughborough University, Loughborough, UK

**Keywords:** EPIDEMIOLOGY, SOCIAL MEDICINE

## Abstract

**Objective:**

Social epidemiology models suggest that socioeconomic status (SES) mobility across the life course affects blood pressure. The aim of this study was to investigate the association between SES change between infancy and adolescence, and blood pressure, in young adults, and the impact of early growth on this relationship.

**Setting:**

Data for this study were obtained from a ‘Birth to Twenty’ cohort in Soweto, Johannesburg, in South Africa.

**Participants:**

The study included 838 Black participants aged 18 years who had household SES measures in infancy and at adolescence, anthropometry at 0, 2, 4 and 18 years of age and blood pressure at the age of 18 years.

**Methods:**

We computed SES change using asset-based household SES in infancy and during adolescence as an exposure variable, and blood pressure and hypertension status as outcomes. Multivariate linear and logistic regressions were used to investigate the associations between SES change from infancy to adolescence, and age, height and sex-specific blood pressure and hypertension prevalence after adjusting for confounders.

**Results:**

Compared to a persistent low SES, an upward SES change from low to high SES tertile between infancy and adolescence was significantly associated with lower systolic blood pressure (SBP) at the age of 18 years (β=−4.85; 95% CI −8.22 to −1.48; p<0.01; r^2^=0.1804) after adjusting for SES in infancy, small-for-gestational-age (SGA) and weight gain. Associations between SES change and SBP were partly explained by weight gain between birth and the age of 18 years. There was no association between SES mobility and diastolic blood pressure, mean arterial pressure or hypertension status.

**Conclusions:**

Our study confirms that upward SES change has a protective effect on SBP by the time participants reach young adulthood. Socioeconomic policies and interventions that address inequality may have the potential to reduce cardiovascular disease burden related to BP in later life.

**Strengths and limitations of this study**

- This present work is a prospective longitudinal cohort study of rigorous design, with the potential to infer causality.
- We employed an objective measure of blood pressure, thereby increasing internal validity of the results.
- One ethnic group comprises the majority of the cohort hence results may not be generalisable to other ethnic groups in South Africa.
- The analytical sample might compromise external validity of the results; however, the study sample was comparable to the excluded group with regard to socioeconomic status in infancy and adolescence, and anthropometry.

## Background

Hypertension, a major public health problem and an independent modifiable risk factor for cardiovascular diseases, is increasingly becoming a problem in low-to-middle income countries (LMICs).^1^ Research has documented that socioeconomic status (SES) influences blood pressure (BP) with low SES being predictive of elevated BP in children^2^ and adults.^3^ ^4^ In addition, early life factors such as birth weight and weight gain may influence the SES change-BP relationship since children from low SES families are likely to be born small and at higher risk of excessive weight gain and high BP.^5^ ^6^

Most of the evidence on social inequalities in BP comes from longitudinal and cross-sectional studies and assumes SES is quite stable over time. However, SES across an individual's lifespan is dynamic in nature especially in societies experiencing sociopolitical transitions, such as South Africa^7^, hence the SES–BP relationship might change even within short periods of time in the early life course.^8^

There has been growing interest in a life course approach to social inequalities in hypertension epidemiology, owing to the evidence that high BP in adulthood evolves from early life; hence the importance of early life environment as a factor influencing the development of hypertension. Life course approaches assume that an individual's health is influenced by dynamic biological and social exposures throughout a life span and that the exposures may not be static over the entire life course.^9^ There are three major conceptual models proposed in life course social epidemiology: social origins (critical periods/latent effect) model, accumulation model and social mobility model.^10^ ^11^

The social origins hypothesis states that early life is a critical period for biological programming where low SES plays a preeminent role in programming health, with children growing up in a low SES environment having raised BP,^12^ independent of their SES in intervening years.^13^ We have previously reported finding no relationship between SES in infancy and BP in this cohort of South African adolescents, in contrast to the social origins hypothesis.^14^ The accumulation model proposes that persisting low SES is detrimental to health. Research on cardiovascular disease risk indicates that low SES in early life has an additive effect on risk factors such as BP.^15^ ^16^ The social mobility model suggests that upward social mobility has a protective effect on hypertension risk while a downward SES change is deleterious to cardiovascular disease risk in adulthood.^17^ ^18^ Hogberg *et al*^19^ reported that intergenerational upward social mobility from low SES was associated with 18% reduction in hypertension risk in a Swedish twin study of 12 030 adults.

The social mobility model has been widely used in life course social epidemiology. However, there is limited literature on social mobility and hypertension, especially among children and adolescents, and most of the studies have concentrated on the intergenerational effect of social mobility on BP, using parental and participants' occupation or education to determine life course SES, or have used later adulthood BP as an outcome. None of the studies adjusted for initial SES and weight gain, making it difficult to disentangle early life SES environmental effects and weight gain from social mobility effects.^11^ ^18–20^

Adolescence is a crucial developmental stage characterised by environmental and social changes, and the onset of hormonal and physiological factors that influence physical health outcomes, including BP.^21^ The studies to date have focused on social mobility in high income countries, where less variability in experiences of SES over the early life course exist compared to the dynamic SES environments of low-income and middle-income countries.^22^

Postapartheid South Africa has been undergoing a rapid social and political transition. The volatility of social environment in the postapartheid era, which has seen improvements in SES in previously disadvantaged Black populations, makes the ‘Birth to Twenty Cohort’ prospective longitudinal cohort a unique and valuable resource to explore the social mobility hypothesis, using BP, which is highly sensitive to changing environments, as an outcome.

This study seeks to test the hypothesis that an upward SES change during childhood and adolescence would be associated with lower BP in early adulthood. Therefore, this study aims to (1) examine the association between SES change and BP and hypertension risk at 18 years of age, and (2) explore whether the SES change–BP relationship is explained by birth outcomes and weight gain between birth and adolescence.

## Methods

### Study design and participants

**Figure 1:**
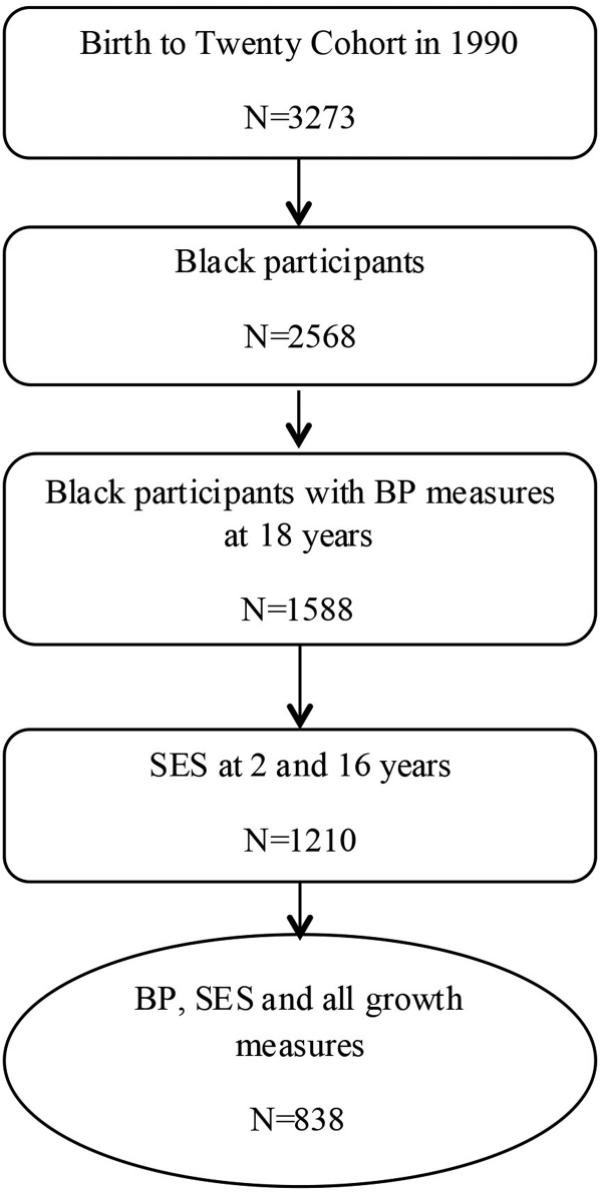
Flow chart of the study population with SES, growth and blood pressure at the age of 18 years. BP, blood pressure; SES, socioeconomic status.

Data for this study came from the ‘Birth to Twenty Cohort’ birth cohort (BT20)—a prospective longitudinal study of children born in Soweto, Johannesburg, South Africa, in 1990. Details of recruitment and enrolment into the cohort study are outlined elsewhere.^23^ Data for this study were collected at birth, and at ages 2, 4, 16 and 18 years. For the purpose of this study, only Black children who had data on BP during late adolescence (18 years), SES data in infancy and during adolescence, birth weight and gestational age, and weight gain in infancy, mid-childhood and from mid-childhood to adolescence, were included in the analysis (n=838). We only selected Black children since they comprise the majority of the BT20 study (figure 1). Ethics approval was obtained from University of Witwatersrand Human Research Ethics Committee (M130556). Informed consent was obtained from caregivers and participants gave their assent at all data collection time points before the participants turned 18 years of age and their consent once they had turned 18 years of age.

### Blood pressure assessment

Blood pressure was measured in triplicate using the Omron M6 (Kyoto, Japan) and an appropriate cuff size with participants in a seated position after an initial 5 min rest, and a 2 min rest between each of the three measurements. An average of the second and third measurements was used for the analyses of systolic BP (SBP), diastolic BP (DBP) and pulse rate. The mean SBP and DBP were used to calculate mean arterial pressure (MAP), using the traditional formula: MAP=((2× diastolic)+systolic)/3.^24^ Hypertension risk was classified using the age, sex and height specific percentiles from the National High Blood Pressure Education Program Working Group on Hypertension control in Children and Adolescence, with hypertension being defined as ≥95th centile and non-hypertension as <95th centile.^25^

### SES change

We used a physical asset-based household SES measures tool in infancy and at 16 years of age, which utilised a validated standardised questionnaire based on the Demographic and Health survey for developing countries (available at: http://www.dhsprogram.com/). The selection of an asset-based household SES was inspired by the notion that assets are more dynamic and sensitive than other measures, such as education and occupation, especially in previously disadvantaged populations undergoing rapid economic and social transition. The physical assets SES measures (eg, television, car and refrigerator) were assessed by asking the caregiver or participant whether they had the asset in question (yes/no). The physical asset scores were computed from all the ‘YES’ answers and were categorised into tertiles: low (1), medium (2) and high (3) for each of the two time points. Thereafter, nine categories of the social mobility model were generated according to the literature and were defined as: low-low (11), low-medium (12), low-high (13), medium-low (21), medium-medium (22), medium-high (23), high-low (31), high-medium (32) and high-high (33).^26^

### Potential confounders and mediators

Sex, gestational age and birth weight were included from data collected at birth. Weight and height at 2, 4 and 18 years were measured using standard procedures. Relative weight gain was defined as weight gain independent of height during infancy, at mid-childhood (2–4 years) and at adolescence to adulthood (4–18 years), and was computed as residuals obtained by regressing current weight on current height, and previous weight and height, to deal with the potential multicollinearity between weight and height.^27^ We also used SES in infancy as a covariate since it was a proxy for early life environment, so that the SES change variable represents a true measure of social mobility. Because BP in children is age, sex and height specific, we adjusted for these three factors in all the models that included SBP, DBP and MAP. To assess alcohol and tobacco use during adolescence, participants 18 years of age were asked whether they had taken alcohol or smoked tobacco in the last month (no/yes).

### Statistical analyses

χ^2^ Tests and t tests were used to describe the study characteristics by sex and hypertension risk for categorical and continuous variables, respectively. Multiple linear regressions were used to assess the association between SES change SBP, DBP and MAP, adjusting for SES in infancy, birth weight and weight gain in infancy, mid-childhood and from mid-childhood to adulthood. We further adjusted the multivariate models for alcohol intake and baseline BP. Additional exploratory models were run for boys and girls separately (results not shown). We also computed the crude and adjusted ORs (and 95% CIs) from logistic regressions for the association between SES change and hypertension risk. The statistical analyses were performed in STATA V.13 with level of significance set at p<0.05 (two tailed).

## Results

### Descriptive statistics

Table 1 shows the study population characteristics by sex and hypertension risk (N=838; 48.0% boys). Boys were heavier at birth and at ages 2 and 4 years and taller at 2, 4 and 18 years than were girls. Systolic BP was significantly higher by 6 mm Hg in boys than in girls; on the contrary, girls had significantly higher DBP than did boys at 18 years of age. There were no sex differences with respect to all SES measures, gestational age, being born SGA, weight at 18 years of age and MAP.

**Table 1:** Study characteristics in infancy and adolescence by sex and blood pressure status at 18 years of age (n=838)

Variables	All	Boys N (%)	Girls N (%)	p Value	Non-Hypertensive N (%)	Hypertensive N (%)	p Value
*Socioeconomic status (exposure)*							
Household SES change between infancy and adolescence, %							
Low-low (ref)	255 (30.4)	133 (33.1)	122 (28.0)	0.522	211 (29.6)	44 (17.3)	0.541
Low-medium	97 (11.6)	45 (11.2)	52 (11.9)		81 (11.3)	16 (12.9)	
Low-high	35 (4.2)	17 (4.2)	18 (4.1)		34 (4.8)	1 (0.81)	
Medium-low	99 (11.8)	41 (10.2)	58 (13.3)		85 (11.9)	14 (11.3)	
Medium-medium	71 (8.5)	32 (8.0)	39 (8.9)		61 (8.5)	10 (8.1)	
Medium-high	43 (5.1)	25 (6.2)	18 (4.1)		38 (5.3)	5 (4.0)	
High-low	78 (9.3)	39 (9.7)	39 (8.9)		67 (9.4)	11 (8.9)	
High-medium	81 (9.7)	37 (9.2)	44 (10.1)		67 (9.4)	14 (12.0)	
High-high	79 (9.4)	33 (8.2)	46 (10.6)		70(9.8)	9 (7.3)	
Total	838	402 (48.0)	436 (52.0)		714 (85.2)	124 (14.8)	
*Participant characteristics*							
In childhood							
Gestational age, weeks (SD)	838	38 (1.7)	38 (1.8)	0.3736	38 (1.7)	38 (1.8)	0.8009
Birth weight, g (SD)	838	3.1 (0.5)	3.0 (0.5)	**<0.01**	3.1 (0.5)	3.1 (0.5)	
SGA, %							
No	743	348 (86.6)	395 (90.6)	0.066	639 (89.5)	104 (83.9)	0.068
Yes	95	54 (13.4)	41 (9.4)		75 (10.5)	20 (16.1)	
Weight at 2 years, kg (SD)	838	11.6 (1.5)	11.3 (1.4)	**0.0177**	11.4 (1.4)	11.5 (1.5)	0.5112
Weight at 4 years, kg (SD)	838	15.6 (1.9)	15.2 (2.0)	**<0.01**	15.3 (2.0)	15.6 (2.0)	0.0884
Height at 2 years, cm (SD)	838	83.4 (3.5)	82.5 (3.2)	**<0.001**	83.0 (3.3)	82.8 (3.5)	0.4768
Height at 4 years, cm (SD)	838	99.1 (3.9)	98.6 (3.8)	**0.0309**	98.8 (3.9)	98.8 (4.0)	0.854
In adolescence							
Age, years (SD)	838	17.8 (0.4)	17.8 (0.4)	0.4521	17.8 (0.4)	17.8 (0.4)	0.2287
Weight at 18 years, kg (SD)	838	59.8 (10.2)	59.3 (12.4)	0.6017	58.7 (10.2)	64.2 (15.5)	**<0.001**
Height at 18 years, cm (SD)	838	170.6 (8.2)	159.6 (6.0)	**<0.001**	165.1 (8.8)	163.5 (9.9)	0.0685
Blood pressure measures at 18 years							
SBP, mm Hg (SD)	838	121 (10.6)	115 (9.5)	**<0.001**	115 (8.5)	131 (11.2)	**<0.001**
DBP, mm Hg (SD)	838	71 (8.5)	72 (8.5)	**0.0410**	70 (6.9)	81 (11.0)	**<0.001**
MAP, mm Hg (SD)	838	87 (8.2)	87 (8.4)	0.1525	85 (6.3)	99 (8.3)	**<0.001**

Bold typeface indicates statistically significant results. Values are presented as mean (SD) computed from a t test for continuous variables or as N (%) for categorical variables obtained from a χ^2^ test and Fischer's Exact test for N<5.

DBP, diastolic blood pressure; MAP, mean arterial pressure; SBP, systolic blood pressure; SES, socioeconomic status; SGA, small-for-gestational age.

Overall, 14.8% of the participants in the study sample were hypertensive (n=124) and 49.1% of these were boys. Table 1 comprises the study characteristics in infancy and adolescence by sex and BP status at the age of 18 years (n=838). Participants who were hypertensive were significantly, 5.5 kg, heavier at the age of 18 years compared to their normotensive counterparts. No major differences in hypertension risk with respect to SES change between infancy and adolescence, birth measures, weight and height in childhood and height at 18 years were observed.

### Determinants of BP and hypertension status

In unadjusted analyses, SBP was significantly associated with change from low to high SES between infancy and adolescence, sex, age, weight and height at 18 years, and relative weight gain independent of height at 0–2 and 4–18 years (see online supplementary appendix 1). DBP was significantly associated with sex (higher in males), age and weight at the age of 18 years and weight gain from ages 4 to 18 years. MAP was predicted by weight and height at 18 years, and weight gain from age 4 to 18 years. Hypertension risk was significantly associated with weight at 18 years and weight gain at ages 2–4 and 4–18 years.

10.1136/bmjopen-2015-008805.supp1 Supplementary data

### Association between SES change and BP and hypertension status

Multiple linear regression analyses of SES change characterised by nine subgroups and age, sex and height-adjusted SBP, DBP and MAP are presented in table 2. SES change from low to high tertile was significantly associated with 4.8 mm Hg lower SBP compared to those who maintained a low SES profile between infancy and adolescence, adjusted for SES in infancy, small-for-gestational-age (SGA) and weight gain between infancy and adulthood. The associations between DBP and MAP, and SES change, were statistically insignificant in all the models.

**Table 2:** Multiple regression models for the relationship between SES change and SBP, DBP and MAP at 18 years of age in urban Black South Africans

Blood pressure measure	SBP						DBP						MAP					
	Model 1 (n=838)			Model 2 (n=838)			Model 1 (n=838)			Model 2 (n=838)			Model 1 (n=838)			Model 2 (n=838)		
Covariates	Β	95% CI	p Value	β	95% CI	p Value	β	95% CI	p Value	β	95% CI	p Value	β	95% CI	p Value	β	95% CI	p Value
SES change																		
Low-low (ref)																		
Low-medium	−0.74	−3.08 to1.60	0.532	−0.38	−2.63 to 1.86	0.737	−0.52	−2.52 to 1.48	0.608	−0.33	−2.32 to 1.66	0.743	−0.62	−2.56 to1.33	0.532	−0.34	−2.24 to 1.55	0.723
Low-high	−5.10	−8.61 to−1.58	**<0.01**	−4.85	−8.22 to −1.48	**<0.01**	−2.41	−5.42 to 0.60	0.117	−2.27	−5.25 to 0.71	0.136	−2.99	−5.91 to 0.07	**0.045**	−2.81	−5.66 to 0.03	0.053
Medium-low	−0.52	−3.52 to 2.48	0.735	−0.69	−3.57 to 2.19	0.639	1.20	−1.37 to 3.77	0.358	1.09	−1.45 to 3.64	0.398	0.44	−2.05 to 2.94	0.725	0.34	−2.09 to 2.77	0.782
Medium-medium	−1.77	−5.01 to1.48	0.285	−2.23	−5.35 to 0.89	0.16	−0.13	−2.91 to 2.64	0.925	−0.34	−3.10 to 2.42	0.811	−1.19	−3.88 to 1.51	0.388	−1.44	−4.07 to 1.19	0.282
Medium-high	−0.90	−4.64 to 2.83	0.634	−1.07	−4.66 to 2.51	0.557	−0.02	−3.22 to 3.18	0.99	−0.15	−3.33 to 3.02	0.925	−0.51	−3.61 to 2.60	0.749	−0.60	−3.63 to 2.43	0.696
High-low	−3.65	−7.79 to 0.48	0.083	−3.93	−7.90 to 0.04	0.062	−1.20	−4.74 to 2.34	0.505	−1.39	−4.90 to 2.13	0.439	−1.81	−5.24 to 1.62	0.302	−1.98	−5.33 to 1.37	0.247
High-medium	−1.38	−5.50 to 2.73	0.51	−2.03	−5.98 to 1.91	0.312	1.36	−2.16 to 4.88	0.448	1.03	−2.45 to 4.53	0.56	0.39	−3.02 to 3.81	0.821	−0.60	−3.39 to 3.27	0.972
High-high	−3.47	−7.84 to 0.90	0.12	−3.41	−7.60 to 0.78	0.34	0.03	−3.71 to 3.77	0.989	0.00	−3.71 to 3.71	1.000	−1.41	−5.04 to 2.23	0.448	−1.35	−4.89 to 2.19	0.456
Sex	−4.03	−5.86 to −2.20	**<0.001**	−4.2	−5.98 to −2.42	**<0.001**	1.94	0.38 to 3.51	**0.015**	1.78	0.21 to 3.37	**0.026**	0.54	−0.98 to 2.06	0.486	0.47	−1.04 to 1.97	0.544
Participant age, years	2.49	0.69 to 4.30	**<0.01**	2.42	0.69 to 4.14	**<0.01**	−1.30	−2.84 to 0.25	0.1	−1.32	−2.85 to 0.21	0.092	−0.08	−1.58 to 1.43	0.921	−0.14	−1.60 to 1.32	0.853
Participant height, cm	0.17	0.06 to 0.28	**<0.01**	0.18	0.08 to 0.29	**<0.01**	0.07	−0.02 to 0.16	0.132	0.07	−0.02 to 0.16	0.131	0.12	0.02 to 0.21	**<0.01**	0.13	0.04 to 0.22	**<0.01**
Household SES in infancy	0.55	−0.46 to 1.55	0.285	0.64	−0.32 to 1.60	0.192	−0.15	−1.01 to 0.70	0.726	−0.10	−0.95 to 0.75	0.818	0.10	−0.73 to 0.93	0.821	0.17	−0.64 to 0.98	0.683
Small-for-Gestational age				0.87	−1.22 to 2.96	0.415				−0.16	−2.01 to 1.69	0.866				0.51	−1.25 to 2.28	0.571
Relative weight gain (0–2 years)				1.06	0.38 to 1.74	**<0.01**				0.49	−0.12 to 1.09	0.114				0.65	0.07 to 1.22	**0.028**
Relative weight gain (2–4 years)				0.65	0.02 to 1.27	**0.044**				0.29	−0.26 to 0.85	0.300				0.62	0.08 to 1.15	**0.023**
Relative weight gain (4–18 years)				2.79	2.12 to 3.47	**<0.001**				1.28	0.68 to 1.87	**<0.001**				1.85	1.28 to 2.42	**<0.001**
Adjusted R² value	0.1053			0.1804			0.0064			0.0260			0.0076			0.0605		

Bold typeface indicates statistically significant results. Model 1: adjusted for sex, current height, age and household SES in infancy.

Model 2: Model 1+growth (SGA, relative weight gain in infancy and mid-childhood).

Baseline BP: SBP at 5 for SBP, DBP at 5 for the DBP and MAP at 5 for the MAP models, accordingly.

DBP, diastolic blood pressure; MAP, mean arterial pressure; SBP, systolic blood pressure; SES, socioeconomic status.

Adjusted logistic regression models (table 3) showed no significant association between SES change from the low-high category and hypertension risk. Relative weight gain at 2–4 and 4–18 years predicted 30% and 66% increased odds of hypertension independent of SES change, SES in infancy, SGA and relative weight gain in infancy.

**Table 3:** Adjusted ORs of being hypertensive at 18 years of age in urban Black South African children (n=838)

	Model 1			Model 2		
Covariates	OR	95% CI	p Value	OR	95% CI	p Value
SES change between infancy and adolescence						
Low-low (ref)	1			1		
Low-medium	0.92	0.48 to 1.72	0.787	0.99	0.51 to 1.88	0.968
Low-high	0.14	0.02 to 1.04	0.055	0.14	0.02 to 1.04	0.055
Medium-low	0.61	0.27 to 1.42	0.255	0.57	0.24 to 1.34	0.197
Medium-medium	0.61	0.25 to 1.52	0.290	0.53	0.21 to 1.36	0.186
Medium-high	0.49	0.16 to 1.50	0.213	0.47	0.15 to 1.48	0.198
High-low	0.51	0.16 to 1.64	0.259	0.46	0.14 to 1.56	0.214
High-medium	0.65	0.21 to 2.02	0.455	0.51	0.16 to 1.65	0.262
High-high	0.38	0.11 to 1.37	0.140	0.36	0.10 to 1.33	0.125
Household SES in infancy	1.14	0.86 to 1.52	0.359	1.20	0.89 to 1.61	0.237
SGA, %				1.33	0.75 to 2.33	0.328
Relative weight gain (0–2 years)				1.18	0.96 to 1.45	0.119
Relative weight gain (2–4 years)				1.31	1.08 to 1.58	**<0.01**
Relative weight gain (4–18 years)				1.65	1.35 to 2.04	**<0.001**
Pseudo R² value	0.0135			0.0630		

Bold typeface indicates statistically significant results. Model 1 adjusted for SES at baseline.

Model 2 model 1+growth (SGA, relative weight gain in infancy and mid-childhood).

SES, socioeconomic status; SGA, small-for-gestational age.

Furthermore, additional multivariate analyses of factors associated with BP and hypertension risk in urban Black South African participants aged 18 years are presented in online supplementary appendix 2. In these associations, adjusting for alcohol intake and baseline BP did not significantly alter the variance explained by the models.

## Discussion

### Main findings

We found that an upward mobility in SES was strongly associated with lower SBP at 18 years of age in contrast to remaining in a low SES profile between infancy and adolescence. This study highlights that the association between upward social mobility and reduced SBP is not fully explained by growth trajectories in relative weight since the association remained significant even after controlling for growth. There was no association between SES change and DBP, MAP and hypertension risk.

### Comparison with other studies

Our results are consistent with previous studies, which reported that upward social mobility is related to reduced BP. The Pitt county study of African American men aged 25–50 years at baseline in 1988 by James *et al*^18^ reported that compared to the stable low SES group between childhood and adulthood, upward SES mobility between childhood and adulthood was associated with a 47% reduction in hypertension risk using education, occupation and employment status to compute life course SES. Childhood SES data were collected retrospectively in this study thereby compromising internal validity of the findings. The Swedish study of twins born between 1926 and 1958 reported 16% lower odds in the upwardly mobile SES group compared to the stable low SES group independent of familial factors.^19^ This study used intergenerational SES measures for life course SES based on the parental and offsprings’ occupation, and self-reported hypertension status, which is prone to information bias.

Contrary to our findings, a US study conducted between 2002 and 2003, reported that children who experience an upward mobility trajectory in SES between 14 and 18 years of age had higher SBP compared to those who remained in the low SES profile. However, the results might have been influenced by the under-representation of low SES children in their study.^13^ Hallal *et al*^28^ found no association between socioeconomic trajectories from birth to 11 years of age and SBP and DBP in 15-year old Brazilian adolescents born in 1993, using household income as an indicator of SES.

### Possible explanation of the findings

Being SGA had no independent effect on the association between SES change and SBP at 18 years implying that postnatal growth might be more important for programming of social gradients in BP than prenatal growth. Social mobility effects on SBP are not fully explained by growth, implying that a dynamic SES environment may influence BP through additional mechanisms. Potential mechanisms through which upward mobility in SES reduces BP have been evaluated, including biobehavioural factors and chronic stress.^29^ An upward mobility in social class might imply that adolescents are protected from negative health behaviour associated with poor households, such as deficient diet, lower levels of physical activity and higher prevalence of tobacco smoking or alcohol intake. However, in this study, adding alcohol use to the models did not alter the associations.

Association between SES change and BP was significant for SBP but not DBP, implying that SBP might be more sensitive to environmental factors compared to DBP. Persistent low SES is a chronic stressor that is related to an increase in sympathetic nervous system reactivity and changes in vasculature, which together raise SBP.^30^ High SBP may be an indicator of vascular dysfunction as a result of progressive stiffening of arterial walls or changes in the vasculature, and it has been reported to be a stronger predictor of hypertension and cardiovascular diseases than DBP.^31^

Sex had a distinct independent relationship with SBP, DBP and hypertension risk. However, when the analyses were stratified by sex, the associations remained significant for boys (results not shown) in the SES change-SBP models only, implying that the protective effect of upward social mobility may be apparent in boys and not girls—but this needs to be further explored with a larger sample size.

### Strengths and limitations

These findings were based on a prospective birth cohort, thereby minimising recall bias and having the potential to establish a causal relationship between life course SES and BP. Asset-based-SES measures are more sensitive for SES compared to education and employment measures, in LMICs, since using schooling years for education might not take into account repeated years,^32^ employment can be informal and transitory, and income and expenditure are notoriously difficult to assess without extensive validation from secondary sources.^33^

In contrast to previous studies on social mobility and hypertension, which used self-reported measures of hypertension, we employed an objective measurement of BP by trained research assistants. Furthermore, the study used both sexes of urban Black South African adolescents from a rapidly transitioning urban environment that can be generalised to other African societies in transition. Sex, age and height-adjusted BP measures were used in the multivariate models since BP in children and adolescents varies according to age, height and sex.^34^ Unlike other studies, we adjusted for covariates to disentangle the effect of early life SES and weight gain on the SES change–BP relationship hence increasing the potential to infer causality.

There are a number of considerations that may pose as limitations. First, we could not include other ethnic groups due to under-representation in the low SES group at the two time points, hence our findings may not be generalisable to the entire South African population. The proportion of hypertensive participants who were in the low-high SES change category was low and this might have resulted in underestimation of the upward social mobility-hypertension risk association resulting in marginal associations. Alcohol intake and tobacco use were self-reported hence we do not rule out reporting bias. There was potential for selection bias in the analytical sample, however, there were no significant differences between the black participants included and those excluded from the study with regard to the key study variables, thereby increasing the potential to generalise these findings.

### Conclusions

Our study adds to the limited body of evidence concerning the protective effect of upward social mobility on BP, and shows an association between SES change in the early life course from birth to adolescence and SBP in early adulthood. There is a need for replication of this study to assess its generalisability in other geographical settings and other ethnic groups. These study findings imply that national social and economic policies introduced in the post-apartheid era that seek to improve quality of life among previously disadvantaged black populations have the potential to reduce cardiovascular disease burden attributed to high BP.
