# Supplementary material for: Association of socioeconomic status change between infancy and adolescence, and blood pressure, in South African young adults: Birth to Twenty Cohort
Source: BMJ Open. 2016 Mar 30;6(3):e008805. doi: 10.1136/bmjopen-2015-008805 (PMC4823398; doi:10.1136/bmjopen-2015-008805)
Supplement: Supplementary data [file bmjopen-2015-008805supp.pdf]

Appendix 1 Bivariate analysis of factors associated with blood pressure and hypertension risk in urban South African black participants aged 18 years (n=838)

	SBP			DBP			MAP			Hypertension risk		
**Exposure variables**	**β**	**95% CI**	**p value**	**β**	**95% CI**	**p value**	**β**	**95% CI**	**p value**	**OR**	**95% CI**	**p value**
**SES change**												
Low-low (ref)										1		
Low-medium	-0.89	-3.34-to 1.56	0.474	-0.39	-2.38 to 1.60	0.702	-0.54	-2.47 to 1.40	0.586	0.95	0.51 to 1.77	0.865
Low-high	-4.94	-8.64-to -1.23	**<0.01**	-2.24	-5.26 to 0.77	0.144	-2.78	-5.71 to 0.14	0.062	0.14	0.02 to 1.06	0.057
Medium-low	-0.13	-2.56 to 2.30	0.916	1.12	-0.86 to 3.10	0.266	0.61	-1.31 to 2.53	0.534	0.79	0.41 to 1.52	0.478
Medium-Medium	-1.39	-4.15 to 1.36	0.321	-0.34	-2.58 to 1.90	0.765	-1.15	-3.33 to 1.03	0.301	0.79	0.37 to 1.65	0.526
Medium-high	0.50	-2.88 to 3.89	0.771	-0.23	-2.99 to 2.52	0.869	-1.16	-2.83 to 2.52	0.909	0.63	0.24 to 1.69	0.361
High-low	-2.29	-4.95 to 0.36	0.091	-1.69	-3.85 to 0.47	0.125	-1.63	-3.73 to 0.47	0.128	0.79	0.38 to 1.61	0.512
High-Medium	-0.23	-2.85 to 2.39	0.865	1.02	-1.11 to 3.15	0.348	0.63	-1.44 to 2.70	0.548	1.00	0.52 to 1.94	0.995
High-high	-2.31	-4.95 to 0.34	0.087	-0.41	-2.56 to 1.74	0.711	-1.21	-3.30 to 0.88	0.256	0.62	0.29t o 1.33	0.216
**Participant characteristics**												
**Childhood**												
Gestational age, weeks	0.01	-0.37 to 0.41	0.943	0.03	-0.28 to 0.35	0.836	0.03	-0.27 to 0.34	0.826	0.97	0.88 to 1.07	0.559
Birth weight, kg	0.40	-0.98 to 1.78	0.568	-0.12	-1.24 to 1.01	0.836	0.00	-1.09 to 1.09	0.999	0.96	0.67 to 1.40	0.861
Small-for-Gestational age(SGA),%												
No(ref)										1		
Yes	2.02	-0.16 to 4.19	0.069	-0.05	-1.83 to 1.74	0.96	0.76	-0.95 to 2.48	0.383	1.56	0.92 to 2.66	0.099
**Adolescence**												
Age, years	2.81	0.98 to 4.65	**<0.001**	-1.1	-2.61 to 0.40	0.15	0.11	-1.35 to 1.56	0.887	1.41	0.86 to 2.30	0.172
Sex												
Boys(ref)										1		
Girls	-6.10	-7.41 to -4.77	**<0.001**	1.19	0.07 to 2.31	**0.04**	-0.81	-1.90 to 0.27	0.142	1.00	0.69 to 1.45	0.99
Alcohol intake												
No										1		
Yes	-1.05	-2.40 to 0.31	0.131	-0.23	-1.38 to 0.93	0.701	-0.50	-1.61 to 0.61	0.378	0.81	0.57 to 1.16	0.259
Smoking												
No										1		
Yes	-1.29	-2.69 to 0.11	0.071	0.93	2.41 to 0.55	0.217	-1.06	-2.69 to 0.57	0.201	0.72	0.44 to 1.19	0.203
Weight at age 18yrs, kg	0.25	0.19 to 0.30	**<0.001**	0.12	0.07 to 0.17	**<0.001**	0.17	0.13 to 0.22	**<0.001**	1.04	1.02 to 1.06[3]	**<0.001**
Height at age 18yrs,cm	0.35	0.27 to 0.42	**<0.001**	0.00	-0.07 to 0.06	0.888	0.10	0.04 to 0.17	**<0.01**	0.99	0.97 to 1.01	0.236

Relative weight gain (0-2years)	0.87	0.15 to 1.59	**0.02**	0.45	-0.14 to 1.04	0.135	0.56	0.00 to 1.13	0.051	1.13	0.94 to 1.38	0.194
Relative weight gain (2-4years)	0.64	-0.02 to 1.30	0.058	0.12	-0.42 to 0.66	0.652	0.48	-0.04 to 1.00	0.068	1.28	1.07 to 1.55	**<0.01**
Relative weight gain (4-18years)	2.56	1.86 to 3.26	**<0.001**	1.29	0.71 to 1.87	**<0.001**	1.77	1.22 to 2.32	**<0.001**	1.59	1.30 to 1.93	**<0.001**

Appendix 2 Additional multivariate analyses of factors associated with blood pressure and hypertension risk in urban South African black participants aged 18 years.

	SBP[1](n=655)				DBP[1](n=655)				MAP[1](n=655)				Hypertension risk[2](n=653)			
	β	95% (CI)		p value	β	95% (CI)		p value	β	95% (CI)		p value	Odds Ratio	95% (CI)		P value
SES change																
Low-low(ref)																
Low-medium	-1.35	-4.19	1.49	0.350	-0.60	-3.12	1.92	0.639	-0.86	-3.27	1.55	0.482	0.61	0.28	1.34	0.215
Low-high	-4.78	-8.92	-0.65	**0.024**	-0.34	-4.02	3.33	0.855	-1.77	-5.28	1.73	0.321	0.27	0.06	1.23	0.091
Medium-low	-0.85	-4.38	2.67	0.634	0.98	-2.16	4.11	0.540	0.35	-2.64	3.34	0.820	0.56	0.22	1.45	0.232
Medium-Medium	-3.64	-7.59	0.32	0.071	-1.69	-5.21	1.82	0.344	-2.37	-5.72	0.99	0.166	0.45	0.15	1.35	0.153
Medium-high	1.07	-3.14	5.28	0.619	1.19	-2.56	4.93	0.533	1.15	-2.43	4.72	0.528	0.64	0.20	2.05	0.458
High-low	-4.28	-9.26	0.71	0.093	-1.06	-5.49	3.38	0.640	-2.15	-6.38	2.08	0.319	0.43	0.10	1.77	0.243
High-Medium	-0.99	-5.89	3.90	0.691	2.81	-1.54	7.16	0.204	1.53	-2.62	5.69	0.469	0.46	0.12	1.75	0.254
High-high	-3.54	-8.76	1.68	0.184	0.99	-3.66	5.63	0.676	-0.52	-4.95	3.91	0.818	0.50	0.12	2.13	0.351
Current participant age, yrs	2.45	0.26	4.64	**0.028**	-1.03	-2.98	0.91	0.298	0.14	-1.71	2.00	0.879				
Current participant height, cm	0.08	-0.05	0.21	0.227	-0.02	-0.13	0.10	0.761	0.01	-0.09	0.12	0.808				
Baseline BP at 5 yrs	0.13	0.07	0.19	**0.000**	0.18	0.09	0.27	**0.000**	0.16	0.08	0.24	**0.000**	1.38	0.84	2.29	0.204
Household SES in infancy	0.29	-0.95	1.53	0.650	-0.39	-1.49	0.71	0.489	-0.16	-1.21	0.89	0.770	1.05	0.74	1.48	0.782
Current alcohol intake	-0.71	-2.31	0.90	0.386	0.13	-1.29	1.56	0.854	-0.16	-1.52	1.20	0.822	0.84	0.54	1.32	0.454
Sex	-4.98	-7.17	-2.78	**0.000**	1.26	-0.71	3.22	0.210	-0.82	-2.69	1.05	0.390				
small for gestational age(SGA)	2.00	-0.45	4.45	0.109	0.45	-1.73	2.63	0.687	0.93	-1.15	3.01	0.379	1.87	1.05	3.32	**0.033**
Relative weight gain (0-2years)	1.19	0.35	2.03	**0.005**	0.53	-0.22	1.27	0.166	0.75	0.04	1.46	**0.039**	1.13	0.90	1.43	0.301
Relative weight gain (2-4years)	0.35	-0.38	1.09	0.348	0.32	-0.33	0.97	0.340	0.32	-0.31	0.94	0.319	1.21	0.98	1.48	0.072
Relative weight gain (4-18years)	3.43	2.62	4.24	**0.000**	1.38	0.65	2.10	**0.000**	2.06	1.37	2.75	**0.000**	1.61	1.28	2.03	**0.000**
	R^2=0.2014				R^2=0.0544				R^2=0.0823				Pseudo R^2=0.0631			

[1]Model adjusted for BP measure and SES at baseline, alcohol intake, height and age at 18yrs, sex, growth (SGA, relative weight gain in infancy and mi-childhood)

[2]Model adjusted for BP measure and SES at baseline, alcohol intake at 18yrs, growth (SGA, relative weight gain in infancy and mi-childhood)
